# Production enhancement of human adipose-derived mesenchymal stem cells by low-intensity ultrasound stimulation

**DOI:** 10.1038/s41598-022-24742-0

**Published:** 2022-12-21

**Authors:** Soohong Min, Yungsun Byeon, Min Kim, Youngjun Lee, Sung-Hoon Lee, Youngjun Lee, Hafiz Muhammad Umer Farooqi, Hong-Ki Lee, Dong-Guk Paeng

**Affiliations:** 1 EHL Bio Inc, Gyeonggi, South Korea; 2 Department of Ocean System Engineering, Jeju National University, Jeju, South Korea; 3 Department of Radiology and Medical Imaging, University of Virginia, Charlottesville, VA USA

**Keywords:** Cell growth, Mesenchymal stem cells, Biomedical engineering, Applied physics

## Abstract

Low-intensity ultrasound (LIUS) enhances the proliferation rate of various mammalian stem cells through mechanical stimulation. This study quantitively finds suitable LIUS stimulation parameters for increasing the proliferation rate of human adipose-derived mesenchymal stem cells (hAdMSCs) for mass production. Various stimulation conditions of LIUS were assessed based on the beam pattern of the ultrasonic transducer and the attenuation of the sound waves. Using optimal LIUS stimulation parameters for enhancing proliferation of hAdMSCs taken from bromodeoxyuridine (BrdU) incorporation assay, long-term culture of hAdMSCs was performed for 16 days. The resultant hAdMSCs were characterized for various biomarkers such as CD34−, CD45−, CD73+, CD95+, CD105+ and cytological staining and a cytokine array assay. LIUS stimulation parameters found for enhancing the hAdMSCs proliferation were the frequency of 5 MHz, an intensity of 300 mWcm^−2^, a duration of 10 min per day, and continuous waves with a 100% duty cycle. The LIUS stimulated hAdMSCs group showed a 3.25-fold increase in the cell number compared to the control group after 16 days of culture. By confirming the effects of quantitatively measured LIUS stimulation on the enhancement of hAdMSCs proliferation, this study may be a foundation for the applications of LIUS stimulation in the industrial-scale production of hAdMSCs.

## Introduction

Stem cell based clinical therapeutics have evoked optimistic expectations for incurable diseases and translational medicine. Mesenchymal stem cells (MSCs) are attracting biomedical scientists to autologous transplantation due to their self-renewal ability, differentiation into other lineages of cells, and proliferating infinitely^1^. MSCs can differentiate into osteoblasts, chondrocytes, and adipocytes, and they are less likely to mutate into cancer cells^2^. Transplanted MSCs are not associated with immunological reactions and pose minimum ethical concern^3–5^. Human adipose-derived mesenchymal stem cells (hAdMSCs) are favored because of their availability and diverse clinical therapeutic applications such as skin regeneration, atopic dermatitis, Crohn’s disease, arthritis, stroke, bone grafting, and Alzheimer’s disease^6–10^. Hence, cell banking systems have already been developed to anticipate the large-scale production of therapeutic hAdMSCs^11^. However, different mammalian stem cells exhibit variable proliferation rates, and their mass production requires strict management of good manufacturing practices (GMP) and production costs. Unfortunately, stem cell therapeutics have a short expiration date for viable cells. In contrast, the fresh supply of stem cells requires meticulous patient scheduling for cell sourcing, cell culture processing, and considerable time to acquire sufficient cell yield for their medical applications. Thus, a technology that can secure a mass amount of the therapeutic stem cells within a set time is crucial.

Several in vitro studies have been conducted to improve the stem cell proliferation rate by optimizing biochemical and physical factors such as supplementation of cell culture media with various growth factors^12,13^ and shear stress that mimics the cell microenvironment^14–19^. These efforts can be divided into two broad categories, e.g., biochemical, and physical methods. Biochemical methods mainly provide cytokines cocktails to the stem cell culture to achieve a high yield of the cells. There are drawbacks associated with this type of method at a commercial level:high costs^20–22^, short expiry dates, and growing ethical concerns due to the sourcing of animal-based growth factors and cytokines^23–25^. Hence, the biotech industry is continuously looking for alternatives to biochemical methods^2,26–28^. Physical methods are getting popular in the stem cell industry as a good alternative to biochemical methods. These methods are non-perishable, reusable, robust and, have high throughput. Hence, these methods dramatically decrease the costs and do not pose any ethical issue. Low-frequency low-magnitude vibrations (LFLMV), low static magnetic fields (LSMF), and surface acoustic wave (SAW) are known to enhance the differentiation potential of adipose-derived mesenchymal stem cells into osteogenic and adipogenic cell lines^29–31^. Similarly, low-intensity ultrasound (LIUS) is a method of mechanical stimulation of the tissue microenvironment; its small amplitude of pressure, high frequency, and pulsed pressure waves catalyze several growth promoter genes of the stimulated cells and promote cellular propagation and differentiation. Unlike LFLM and LSMF, LIUS is considered the safest and most highly scalable method of advanced cell culture research. LIUS has a long history of clinical applications and has been employed in many successful clinical trials recently; it has been applied to study the differentiation of stem cells^32–40^. Ultrasound's altered physical pressure can enhance cell proliferation via integrin activation^41–43^. It may resolve the limitations of the cell therapy industry by saving cell culture time, human resources, and production costs. Considering the cell size, sound wave frequency, intensity, and duration are critical factors for LIUS stimulation^44^. Choosing the appropriate ultrasound intensity for efficient stimulation without damaging cells is peculiar to each cell type. The duration of a pulse wave should be considered in controlling average temporal intensity. A continuous or pulsed ultrasound wave may harm the cell culture^34^. Similarly, sonication duration and interval would also affect cell proliferation. Thus, the optimum combination of the parameters of LIUS is crucial to achieving the maximum yield from cell culture.

In various studies, cells subjected to LIUS stimulation exhibited either increased proliferation rates or survival rates under certain conditions. However, the optimization of the LIUS stimulation parameters for various cell types has not been achieved yet^32–35^. Thus, the application of LIUS stimulation for enhanced stem cell proliferation requires an accurate and quantitative selection of multiple parameters such as the frequency suitable for each cell size, the effective intensity without inflicting cellular damage, the stimulation time, and the mode of ultrasound radiation. Ultrasound intensity applied to different types of cells should be adjusted according to the material and thickness of the cell culture flask. The beam pattern in a non-uniform near-field of the ultrasound transducer must also be measured for LIUS stimulation conditions. This study aims to increase the proliferation of hAdMSCs by LIUS stimulation with an accurately measured ultrasound field during the 16-day cell culture. Different parameters of LIUS stimulation for hAdMSCs culture were adopted, and the resultant cell yield was characterized for hAdMSCs specific biomarkers.

## Results

### Cell proliferation rates with different ultrasound parameters

**Figure 1:**
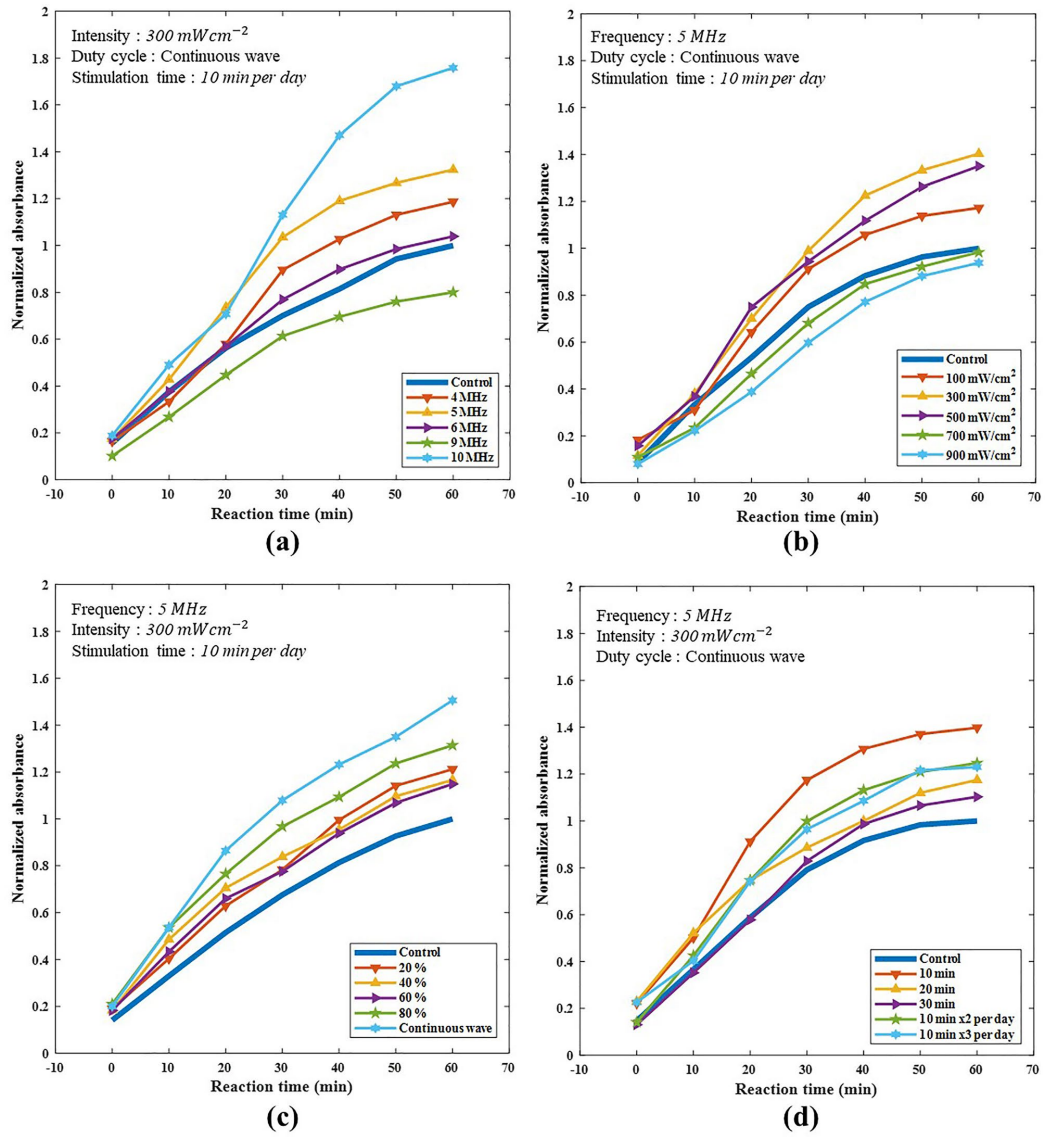
Proliferation rates of hAdMSCs based on various LIUS stimulation parameters using BrdU incorporation assay. **(a)** The graph shows the effect of LIUS stimulation frequency on the proliferation of hAdMSCs. The frequency of 4 MHz, 5 MHz, 6 MHz, 9 MHz, and 10 MHz was applied for 10 min per day. The intensity level was fixed at 300 mWcm^−2^. **(b)** The graph represents the effect of LIUS stimulation intensity on the proliferation of hAdMSCs. The intensity of 100 mWcm^−2^, 300 mWcm^−2^, 500 mWcm^−2^, 700 mWcm^−2^ and 900 mWcm^−2^ were applied for 10 min per day. The frequency was fixed at 5 MHz. **(c)** The graph shows the effect of the LIUS stimulation duty cycle on the proliferation of hAdMSCs. The duty cycle was 20%, 40%, 60%, 80%, and continuous-wave for 10 min per day. The intensity level and frequency were fixed at mWcm^−2^ and 5 MHz. **(d)** The graph shows the effect of the LIUS stimulation time on the proliferation of hAdMSCs. The time durations selected were 10 min per day, 20 min per day, 30 min per day, twice with 10 min per day (Downtime: 12 h), and three time with 10 min per day (Downtime: 8 h). The intensity level and frequency were fixed at 300 mWcm^−2^ and 5 MHz.

BrdU incorporation assay was carried out to investigate the effects of LIUS stimulation parameters on hAdMSCs proliferation rate, and the endpoint absorbance values were determined for up to 90 min. However, the assay results were optimal at 60 min and absorbance was found to be saturated after that. Figure 1 represents the impact of frequency, intensity, duty cycle, and LIUS stimulation duration on hAdMSCs proliferation, where several parameters were set to identify the optimal LIUS stimulation conditions. All the experimental results were normalized against the absorbance of the control group after 60 min of fluorescence reaction. As shown in Fig. 1a, hAdMSCs proliferation at a frequency of 10 MHz produced the highest normalized absorbance of 1.76 compared to the control group. While the normalized absorbances in the cases of frequencies of 5 MHz, 4 MHz, 6 MHz, and 9 MHz, normalized absorbance values were 1.32, 1.19, 1.07, and 0.8, respectively. Although the normalized absorbance at the frequency of 10 MHz was the highest, the frequency of 5 MHz with a standard deviation of 0.23 was selected because the standard deviation of 0.47 at 10 MHz was relatively high. The normalized absorbance at the frequency of 9 MHz was 0.8 due to the heat generated systemically on the transducer surface at an intensity of 300 mWcm^−2^ as shown in Fig. 1a. The hAdMSCs proliferation rate according to the intensity of the ultrasound signal has been presented in Fig. 1b. The normalized absorbance of the wave intensity at 300 mWcm^−2^ was the highest (1.40), whereas at 100 mWcm^−2^ and 500 mWcm^−2^ it was recorded as 1.35 and 1.17, respectively. The heat generation at the transducer surface at higher wave intensities of 700 mWcm^−2^ and 900 mWcm^−2^ gave rise to the lower normalized absorbance. The change in normalized absorbance based on the duty cycle (0–100%) is described in Fig. 1c, where no change in normalized absorbance was observed from 20 to 60%, although it increased to 1.31 at 80% and 1.51 at 100%. LIUS stimulation of hAdMSCs culture for 10 min each day resulted in the highest normalized absorbance of 1.34. While LIUS stimulation twice or thrice a day for 10 min resulted in the normalized absorbance of 1.25 and 1.23, respectively, suggesting that the proliferation rate decreased as the stimulation time increased to 20 and 30 min, as shown in Fig. 1d.

Table 1 summarizes all experimented parameters' normalized absorbance values and standard deviation (SD). Based on these experimental data, appropriate conditions with the maximum stability and the highest proliferation rate of hAdMSCs under LIUS stimulation were frequency of 5 MHz, the intensity of 300 mWcm^−2^, continuous-wave, and 10 min/day.

**Table 1:** Average of normalized absorbance values and standard deviation (SD) based on BrdU incoporation assay.

Frequency (MHz)	Control	4	5	6	9	10
Normalized absorbance	1.00	1.19	1.32	1.04	0.80	1.76
SD	0.12	0.22	0.23	0.21	0.18	0.47

Intensity (${\mathrm{mW}/\mathrm{cm}}^{2}$)	Control	100	300	500	700	900
Normalized absorbance	1.00	1.17	1.40	1.35	0.98	0.94
SD	0.28	0.19	0.22	0.18	0.15	0.18

Duty cycle (%, period = 10 ms)	Control	20	40	60	80	Continuous-wave
Normalized absorbance	1.00	1.21	1.17	1.15	1.31	1.51
SD	0.11	0.06	0.14	0.27	0.23	0.15

Stimulation time (Min)	Control	10	20	30	10 × 2	10 × 3
Normalized absorbance	1.00	1.34	1.17	1.03	1.25	1.23
SD	0.15	0.20	0.15	0.12	0.10	0.18

### Increased number of cells during 16-day culturing

**Figure 2:**
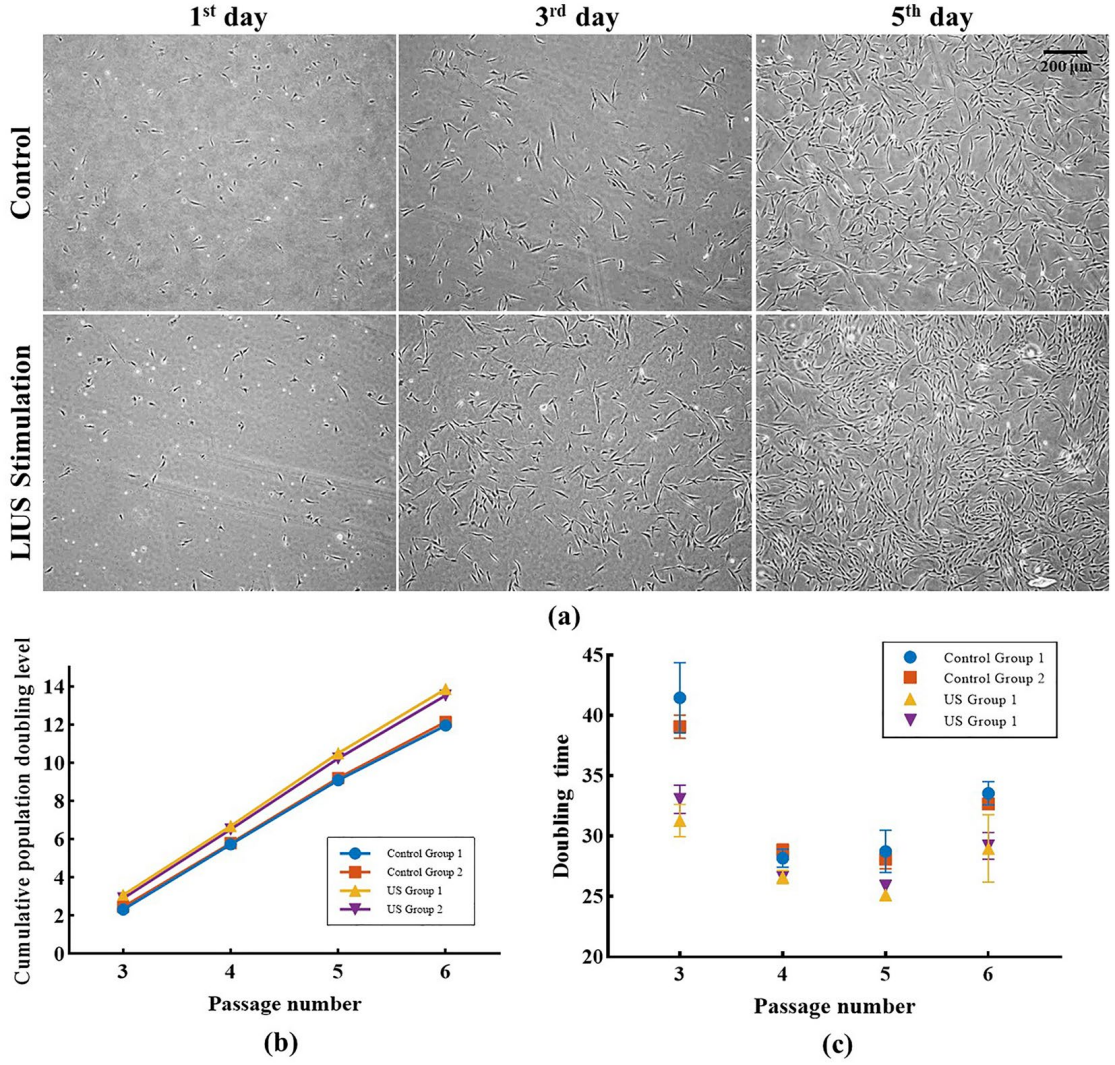
The difference in hAdMSCs growth between the control groups and LIUS stimulated groups. **(a)** Microscopic images of hAdMSCs culture flask of the control group and the LIUS stimulated group at passage 2 with the cell seeding density of 2300 cells/cm^2^. The inverted microscopic images were taken on the 1st, 3rd, and 5th days of the hAdMSCs culture. **(b)** Cumulative population doubling level (CPDL) of control and LIUS stimulated groups of hAdMSCs culture from passage 3 to passage 6. **(c)** Variation in the doubling time of hAdMSCs during cell culture control groups, and LIUS stimulated groups from passage 3 to passage 6.

Long-term subculturing of hAdMSCs was conducted with the set parameters of LIUS stimulation based on the results of the BrdU incorporation assay. Cell numbers in LIUS stimulated groups of passage 2 were higher than those in the control groups, as shown in Fig. 2a. The number of hAdMSCs in control groups (C1 and C2) at passage 3 increased 5.28-fold compared to the seeding number of cells. In passages 4, 5, and 6, the average number of cells increased by 10.34, 10.51, and 7.48-fold, respectively. In the case of LIUS stimulated groups (U1 and U2), the average number of cells compared with the seeding number of cells increased by 8.35, 12.20, 13.35, and 10.07-fold in passages 3, 4, 5, and 6, respectively. The summary of the number of hAdMSCs after culture compared with the number of seeding cells is presented in Table 2.

**Table 2:** Results of cell culture experiment.

	Seeding cells	Passage 3	Passage 4	Passage 5	Passage 6
C1 (× ${10}^{6}$)	0.060	0.303	0.638	0.618	0.437
C2 (× ${10}^{6}$)	0.060	0.330	0.603	0.643	0.460
Average (× ${10}^{6}$)	0.060	0.317	0.621	0.631	0.448
Multiples of seeding cells	1.00	5.28	10.34	10.51	7.48
U1 (× ${10}^{6}$)	0.060	0.507	0.732	0.848	0.618
U2 (× ${10}^{6}$)	0.060	0.451	0.732	0.787	0.590
Average (× ${10}^{6}$)	0.060	0.501	0.732	0.818	0.604
Multiples of seeding cells	1.00	8.35	12.20	13.63	10.07

The initial number of cells in all passages is 60,000 cells. The number of cells for each passage and the increased cell fold was summarized.

### Doubling time

Cumulative population doubling level (CPDL) and doubling time of hAdMSCs culture were calculated based on the subculture cycle and the number of cells in each subculture. In Fig. 2b, the CPDL of the control group increased from 2.32 to 12.15, while the CPDL of the LIUS stimulated group increased from 2.91 to 13.87. The difference in the doubling time of hAdMSCs between the control and LIUS stimulated groups in passage 2 was the highest, ranging from 6 to 10 h. While in passage 3 it was the lowest at 1.5 to 2.7 h, as presented in Fig. 2c. The increase in the doubling time of passage 3 to passage 5 suggests the efficacy of the LIUS stimulation on hAdMSCs culture. The control group showed an average doubling time of 32.63 h, while the LIUS stimulated group exhibited a doubling time of 28.19 h, which is 4.44 h less than the control group. A t-test was applied to determine the similarity metrics between the control and LIUS stimulated groups; the null hypothesis was rejected at the significance level of 5% with a p-value of 0.0485. These results suggest that LIUS stimulation might positively impact reducing the incubation time of hAdMSCs subculture to passage with a shorter doubling time.

### Molecular characterization and phenotyping of LIUS stimulated AdMSCs

**Figure 3:**
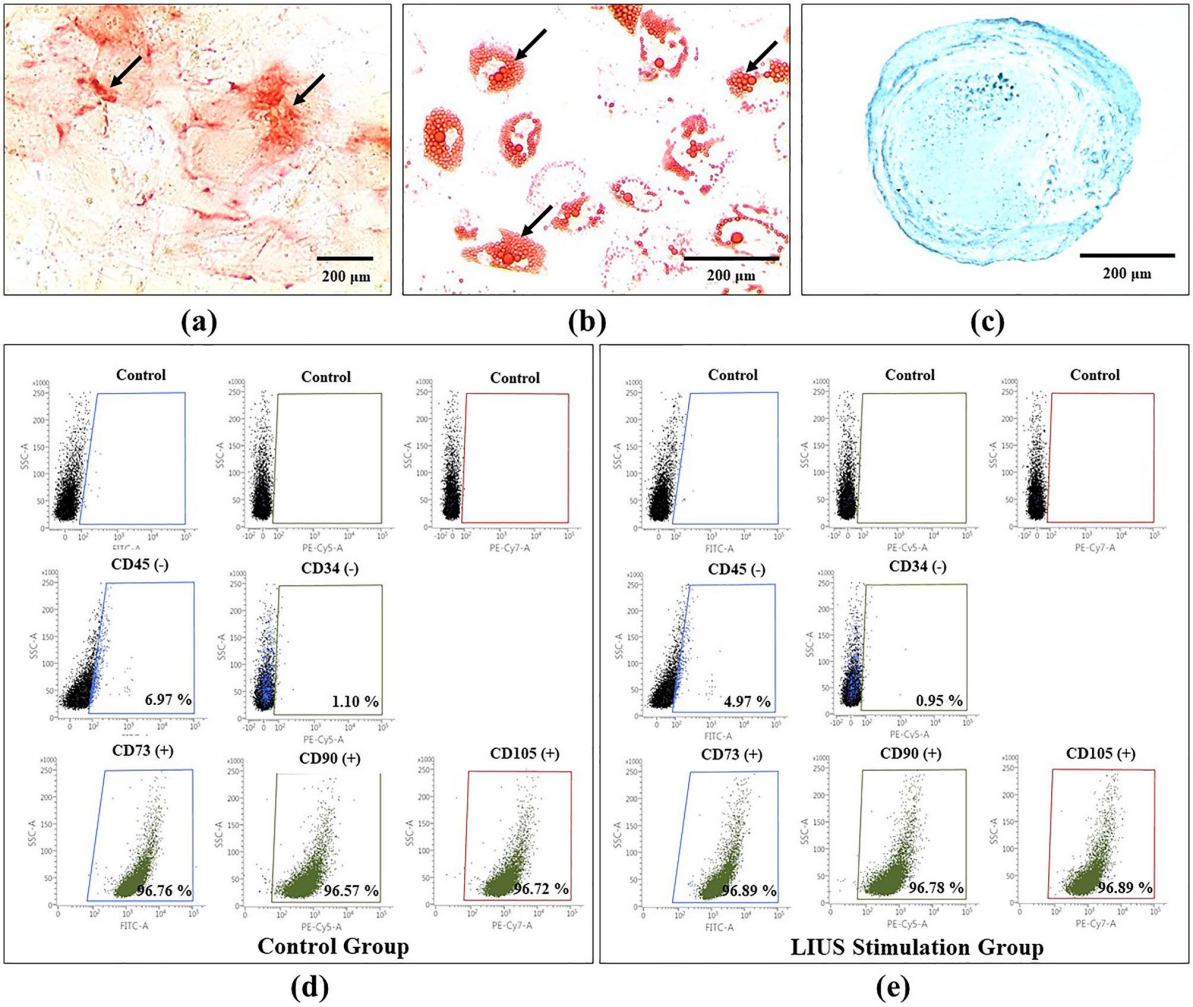
The differentiation potential and the characterization of the control and LIUS stimulation groups of AdMSCs. **(a)** The osteogenic differentiation of hAdMSCs was verified by detecting extracellular calcium deposition. The microscopic image of the Alizarin red S staining indicates the presence of extracellular calcium by red nodules (black arrowheads), **(b)** Oil Red O staining of AdMSCs revealed the presence of intracellular lipid vacuoles, apparent as intracellular red and orange droplets (black arrowheads) which is indicative of adipogenic differentiation of AdMSCs. **(c)** Alcian Blue staining microscopic image for evaluating the chondrogenic differentiation potential of AdMSCs. **(d**) Dot plots from the flow cytometric analysis of the control group with AdMSCs specific negative markers (CD34, CD45) and positive markers (CD73, CD95, and CD105). **(e)** Dot plots from the flow cytometric analysis of the LIUS stimulated group with AdMSCs specific negative markers (CD34, CD45) and positive markers (CD73, CD95, and CD105).

Fluorescence-activated cell sorting (FACS) was performed to identify the AdMSCs specific biomarkers. LIUS stimulated AdMSCs and the control group AdMSCs were positive for CD73, CD, 95, and CD105. In comparison, both groups of the cells showed the absence of CD34 and CD45 on their cell surface. Dot plots of the FACS analysis are presented in Fig. 3d, e. Hence, no significant difference in the characteristics of the LIUS stimulated AdMSCs and the control group AdMSCs was present. Additionally, the cell viability data of LIUS stimulated AdMSCs and control group AdMSCs is presented in Table 3, which shows more than 95% viability in both groups. It can be assumed that LIUS stimulation did not have adverse effects on AdMSCs.

**Table 3:** Cell viability of control groups (C1, C2) and LIUS stimulation groups (U1, U2).

%	Passage 3	Passage 4	Passage 5	Passage 6
C1	98.10	97.50	98.70	98.50
C2	98.38	98.12	98.13	98.05
U1	95.43	99.03	98.28	98.63
U2	97.98	98.60	98.73	97.55

Cell viability in each passage exceeded 95%, confirming that LIUS stimulation did not decrease cell viability.

LIUS stimulated AdMSCs were further differentiated into osteocytes, adipocytes, and chondrocytes to confirm their differentiation potential. Osteocytes exhibit characteristic phenotypic features of extracellular calcium deposition. Hence, LIUS stimulated AdMSCs were differentiated into osteocytes, and Alizarin Red S staining was performed. As shown in Fig. 3a, the resultant osteoblasts stained red and orange due to extracellular calcium deposition. Likewise, the adipogenic differentiation of LIUS stimulated AdMSCs was carried out, and the adipocytes were examined for the presence of intracellular fats. The Oil Red O staining demonstrated the intracellular fat deposition. Characteristic intracellular lipid vacuoles were found in the LIUS stimulated AdMSCs derived adipocytes, as shown in Fig. 3b. Similarly, chondrogenic differentiation of LIUS stimulated AdMSCs was confirmed by staining LIUS stimulated AdMSCs derived chondrocytes with Alcian blue dye. The chondrocytes successfully demonstrate the extracellular matrix presence, as presented in Fig. 3c.

### Evaluation of differentially expressed cytokine release by LIUS stimulated AdMSCs and the control group AdMSCs

The release of various cytokines is hypothesized to be a characteristic feature of stem cells. AdMSCs are known to produce hundreds of cytokines during the process of propagation. Hence, a human cytokine array was conducted to estimate and compare the differentially expressed cytokine release by LIUS stimulated AdMSCs and the control group AdMSCs. LIUS stimulated AdMSCs produced 0.5 to 2.5-(Log2) fold more cytokines than the control group AdMSCs.

## Discussion

In this study, LIUS stimulation, one of the various mechanical stimulation methods, was applied and evaluated to improve the proliferation of hAdMSCs. BrdU incorporation assay was utilized to determine the optimal ultrasound parameters for the hAdMSCs stimulation. As a result, a frequency of 5 MHz, an intensity of 300 mWcm^−2^, a duration of 10 min/day, and a continuous wave were established as the optimal conditions. The simulation area and the effectiveness of the ultrasound transducer were quantitatively evaluated by measuring the beam pattern. The attenuation of ultrasound transmitted through the Petri-dish was also experimentally confirmed. With the set parameters, hAdMSCs were cultured from passage 2 to passage 6 for 16 days. Results demonstrate that LIUS stimulation increased the hAdMSCs population by 3.25-fold compared to the control group. Application of LIUS to hAdMSCs culture induced increased cell proliferation rate and preserved characteristics of stem cells. Cell viability in each passage exceeded 95%, indicating that the LIUS stimulation did not impart any negative impact. It is crucial in the cell therapeutics industry and can save the hectic patient scheduling for cell isolation and the lengthy production process involving human resources and expenses.

In various studies, cells subjected to LIUS stimulation exhibited either increased proliferation rates or survival rates under certain conditions. However, the optimization of the LIUS stimulation parameters for various cell types has not been achieved yet. Several scientists have reported the correlation between LIUS stimulation and cell proliferation rate. Li Yang et al. demonstrated that LIUS stimulation at a frequency of 1 MHz, an intensity of 500 or 1500 mWcm^−2^, and a duration of 10 min for 2 to 4 days improved the proliferation rate of neural crest stem cells based on ethynyldeoxyuridine (EdU) assay^32^. Another study reported that LIUS stimulation at a frequency of 1 MHz and an intensity of 100, 200, or 300 mWcm^−2^ for 10 min/day over seven days increased the survival rate of human articular chondrocytes by increasing the collagen type II and proteoglycan in the extracellular space^33^. These results demonstrate that LIUS can increase the proliferation rate and stimulate the production of extracellular matrix proteins, which is vital for stem cell propagation and differentiation. Zeinab et al. evaluated LIUS stimulation on guinea pig sourced AdMSCs at a frequency of 40 kHz and an intensity of 120–350 mWcm^−2^ and revealed better cell proliferation than the continuous LIUS stimulation at an intensity of 230 mWcm^−2^^34^. Similarly, dental pulp stem cells (DPSCs), bone marrow stem cells (BMSCs), and periodontal ligament stem cells (PDLSC) were stimulated with LIUS at a frequency of 1 MHz, DPSCs and BMSCs exhibited the highest proliferation rate at an intensity of 750 mWcm^−2^ with 5 min of stimulation, while PDLSCs showed the maximum proliferation at an intensity of 250 mWcm^−2^ with 5 min of stimulation^35^. Because stimulation conditions suitable for proliferation improvement are different for each cell type, the ultrasound stimulation conditions identified in this study can be helpful when applying ultrasound stimulation to enhance proliferation of hAdMSCs.

**Figure 4:**
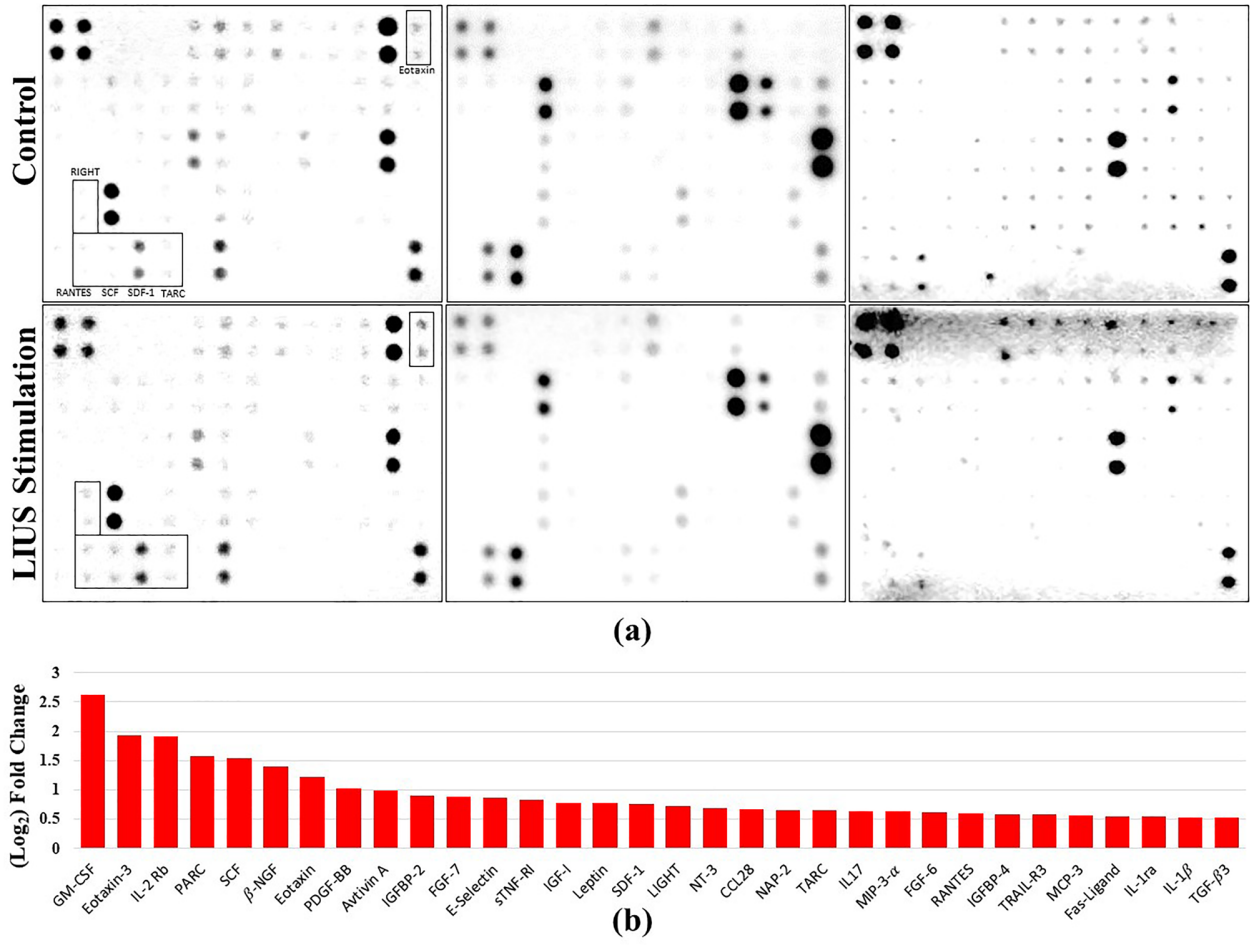
Cytokine expression in LIUS stimulated AdMSCs, and the control group AdMSCs. **(a)** Actual images of human cytokine array membrane with 174 targets. **(b)** A bar chart representing the amount of cytokine release of LIUS stimulated AdMSCs, and the control group AdMSCs from passage 6. Log2 fold change was confirmed using the intensity value and plotted cytokines improved by 0.5 or more.

A cytokines array assay was performed to detect LIUS stimulation’s effects on the release of cytokines by hAdMSCs. The release of 174 cytokines was evaluated and compared with the control group. Figure 4. illustrates lists of 32 cytokines that had log2 fold change values of 0.5 or higher. The release of various cytokines was increased upon LIUS stimulation, and some of them are related to increased cell proliferation rate, GM-CSF^45^ and SCF^46^. Further investigations are necessary to understand the mechanism and relationship between LIUS stimulation and an increase in cell proliferation rate, which may be due to the release of a higher amount of various cytokines.

The generation of the same intensity of 300 mWcm^−2^ from several frequencies using two transducers with different frequency responses requires the adjustment of input voltages. Hence, frequencies outside the resonance frequency of each transducer (4 and 6 MHz, 9 MHz) require an increased amount of voltage, leading to heat generation in the electrical system and eventually on the surface of the ultrasound transducer. Continuous-wave generates higher heat in contrast to a pulsed wave and contributes to a similar or lower proliferation rate than the control rate at frequencies of 6 and 9 MHz and at an intensity of 700 mWcm^−2^ and 900 mWcm^−2^ as shown in Fig. 1a,b. Temperature measurements under these conditions were 38.5 °C, 42 °C (Intensity: 700 mWcm^−2^), 42 °C (Frequency: 9 MHz), and 45 °C, which was substantially higher than the incubation temperature of 37 °C as presented in supplementary information Table S1. Rectifying these anomalies requires constructing an ultrasound transducer and its systemic supplementation by matching the impedance of an electronic module or installing an insulator between the transducer and the cell culture Petri-dish. The frequency of 5 MHz was selected due to the relatively higher stability and constancy. Although the cell proliferation rate at 10 MHz was the highest, its variation was high. With the optimized electronic system, the higher frequencies, including the resonant frequency similar to cell size and cell layer, need to be further investigated in the future. The maximum thickness of the cellular substrate of hAdMSC increased from 10 to 30 μm to about 100 μm. Considering that the wavelength of 5 MHz was 300 μm, the LIUS stimulation efficiency increased at a higher frequency.

Continuous-wave had the highest proliferation rate, as shown in Fig. 1c. Still, cells showed a higher proliferation rate when treated for 10 min each day compared to longer durations, thereby establishing that the proliferation efficiency is not directly proportional to the exposure time. However, the reasons for the higher proliferation rate at 10 min each day need to be explored to establish the optimal diurnal duration. Continuous-wave quickly generates heat in the electronic system and transducer at higher intensity, suggesting the need for selecting the most appropriate wave intensity with a sufficient sonication duration for the optimum cell culture conditions without generating excessive heat.

Cell characteristics, including proliferation rate, might differ between individual cases. However, this study assumed that the allogenic line of hAdMSCs would share the optimal parameter of LIUS stimulation, although it might vary in the degree of increase in the proliferation rate according to each LIUS stimulation parameter. Therefore, a passage of hAdMSCs was divided into two lines; one line was employed for all BrdU incorporation assays. The other was utilized for the whole-cell culture experiment from passages 2 to 6.

**Figure 5:**
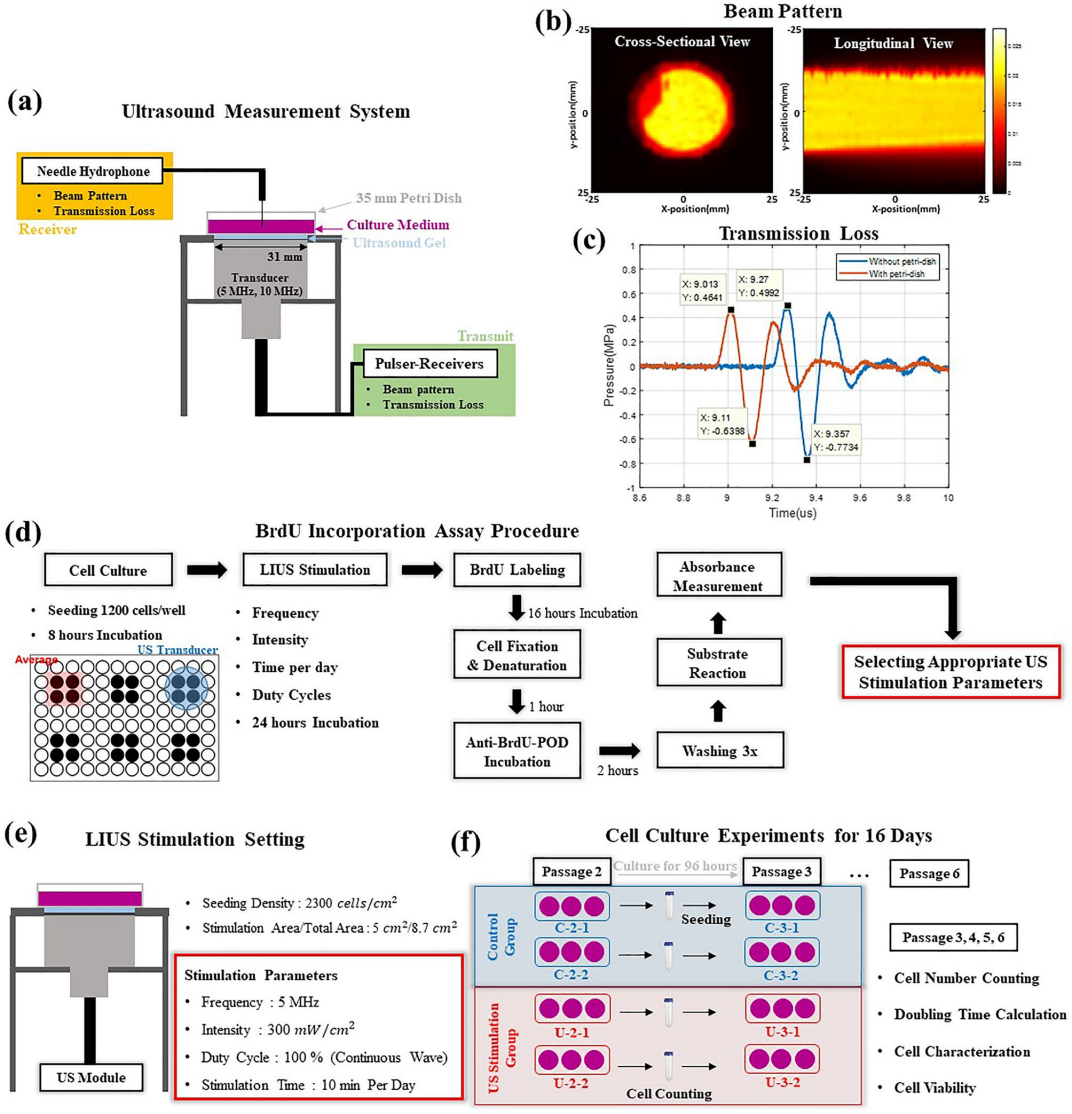
Schematic representation of research method. **(a)** Illustration of the ultrasound measurement system. Pulser-receiver (transmission unit) (5900PR, Panametrics, USA), hydrophone (reception unit) (0.2 mm needle hydrophone, Precision Acoustics, UK), and LIUS transducers with resonance frequencies of 5 MHz (V307-SU, OLYMPUS, USA) and 10 MHz (V322-SU, OLYMPUS, USA). **(b)** Beam pattern of 5 MHz ultrasound transducer, cross-sectional and longitudinal view. **(c)** Ultrasound signal measured using the needle hydrophone with or without the Petri-Dish. When the ultrasound signal passes through the Petri-Dish, the sound speed was increased to receive the signal earlier while the peak-to-peak sound pressure was decreased. **(d)** Block diagram outlining the procedure of LIUS stimulation and BrdU incorporation assay. Cells were seeded at a density of 1200 cells/well. LIUS was applied after 8 h of incubation. The BrdU incorporation assay was conducted 24 h after LIUS stimulation to determine the hAdMSC proliferation rate depending on ultrasound parameters such as frequency, intensity, duty cycle, and sonication time per day. BrdU was incorporated into the newly synthesized DNA strand, replacing thymidine in the DNA of the proliferating cells during BrdU labeling for 16 h. Anti-BrdU-PODs were added after cell fixation. Cells were washed thrice, and a substrate solution was added, followed by the absorbance measurement using a microplate reader. A 96-well plate used for the experiment is illustrated on the lower left of the diagram. Experiments were conducted in four wells in each experimental group covering the area of the US transducer. **(e)** LIUS apparatus for hAdMSCs stimulation in a Petri-Dish. During the BrdU assay, cells were stimulated under appropriate ultrasound (mentioned in the figure). **(f)** Outline of cell culture experiments during 16-day (subculturing steps from passages 2 to 6). Each passage was conducted after 96 h of incubation. The cell number counting, doubling time, MSCs characterization, and survival rates were recorded.

The diameter of the Petri-Dish used in this study was 35 mm, a little wider than the diameter of the transducer aperture (31 mm). The cell culture area used for LIUS stimulation was 25.4 mm in diameter, as shown in Fig. 5b. Thus, 52% of the whole Petri-Dish area was stimulated, suggesting that the proliferation rate might increase by using a transducer covering the entire surface area. Mammalian cells are usually cultured in 75,175 ${\mathrm{mm}}^{2}$ flasks, much wider than the Petri-Dish used in this experiment. Hence, there is a need to design novel bioreactors for LIUS stimulation-based cell culture using a 3-D incubation method for industrial-scale mass production of the hAdMSCs. In this study, optimal parameters of LIUS stimulation that affect proliferation rate without damaging that cell are introduced and confirmed. It may also culminate that the application of LIUS in connection with newly developed bioreactors may solve problems of long cell culture time and high manufacturing costs in the cell therapeutics industry.

## Materials and methods

### Ultrasound measurement system

One of the essential components in cell culture studies exposed to LIUS stimulation is the quantitative evaluation of the ultrasound field. LIUS transducers with an element size of 2.54 cm had resonance frequencies of 5 MHz (V307-SU, OLYMPUS, USA) and 10 MHz (V322-SU, OLYMPUS, USA) were employed in the current study. A pulse-receiver (5900PR, Panametrics, USA) and a hydrophone (0.2 mm needle hydrophone, Precision Acoustics, UK) were utilized in the beam pattern measurement to establish the stimulation area and attenuation coefficient of ultrasound signal passing through a Petri-Dish. Settings of the pulse-receiver were pulse repetition frequency of 100 Hz, damping resistance of 50 Ω yielding 20 dB, attenuation of 0 dB at an energy level of 12.5 μJ, and the filter frequency of 10 to 20 MHz. In LIUS stimulation of hAdMSCs, a US module (Research model, MediFUS, South Korea) was adopted as a transmission unit to adjust frequency, intensity, and duty cycle. By quantifying the spatial average of the average temporal intensity depending on the module input voltage with a power meter (UPM-DT-10PA, Ohmic Instruments, USA), desired intensities at each frequency were obtained and used for LIUS stimulation. An ultrasound gel layer of 3 mm (Sono Jelly, Care Pharm, South Korea) was used to avoid an air layer between the surface of a US transducer and the Petri-Dish to prevent cellular damage caused by the heat produced from the surface of the US transducer. Figure 5a illustrates the settings of ultrasound measurement.

### Beam pattern

Cross-sectional and longitudinal views of a 5 MHz transducer with a defective area have been presented in Fig. 5b. Experiments were conducted assuming that a defective area of less than 10% of the entire area is negligible. The longitudinal view was measured from 3 to 80 mm along the beam direction. The distance from the transducer surface to the cells was 5 mm (3 mm for the ultrasound gel layer, 1 mm for the Petri-dish layer, and 1 mm for the hydrophone measuring point). The sound pressure was effectively transmitted through the 5 mm distance along the x-axis. Additionally, the beam pattern of the 10 MHz transducers was measured. There was no defective area, and an even sound pressure field was confirmed. Thus, the cell culture area stimulated by LIUS was identified as approximately 52% of the area of the Petri-Dish (35 mm diameter).

### Transmission coefficient

1 $$Z(Acoustic \,impedance)= \rho * c$$

2 $$k\left(Wave \,number\right)= \frac{\omega }{c} = \frac{2\pi f}{c}$$

3 $$T\left(Transmission \,coefficient \,of \,sound \,intensity \,at \,3 \,layers\right)=\frac{4}{2+\left(\frac{{Z}_{3}}{{Z}_{1}}+\frac{{Z}_{1}}{{Z}_{3}}\right){cos}^{2}\left({k}_{2}d\right)+\left(\frac{{{Z}_{2}}^{2}}{{Z}_{1}{Z}_{3}}+\frac{{Z}_{1}{Z}_{3}}{{{Z}_{2}}^{2}}\right){sin}^{2}\left({k}_{2}d\right)}$$

4 $$I(Intensity)=\frac{{P}^{2}}{2Z}$$

Transmission loss and attenuation are necessary to establish the attenuation of sound waves radiating from the transducer to hAdMSCs. Based on the thickness of the Petri-dish and the wave number provided, the theoretical acoustic intensity transmission coefficient was calculated to be 0.82 using Eq. 3^34^. Equation (3) considers sound wave transmission through three layers: the ultrasonic gel, the Petri-dish, and the cell culture medium. Z is the acoustic impedance of each layer and the product of the medium density $(\rho )$ and speed of sound $(\mathrm{c})$ as indicated in Eq. 1. $k$ denotes the wavenumber, which is obtained by dividing the angular frequency $(\omega )$ by speed of sound as shown in Eq. 2. ${Z}_{1}$, ${Z}_{2}$, and ${Z}_{3}$ represent the impedance of ultrasonic gel, Petri-dish, and cell culture medium, respectively. This study regarded the cell culture medium and the ultrasonic gel as water. ${\rho }_{1}$, ${\rho }_{2}$, and ${\rho }_{3}$ are 1000, 1500, and 1000 $\mathrm{kg}/{\mathrm{m}}^{3}$, respectively. ${c}_{1}$, ${c}_{2}$, are ${c}_{3}$ are 1500, 2257, and 1500 m/s, respectively. Substituting these values Z_1,3_ = 1.5 × 10^6^ rayls and Z_2_ = 2.37 × 10^6^ rayls were obtained. Figure 5c displays that the ratio of the peak-to-peak sound pressure with and without the Petri-dish (1.2726 and 1.1039 Mpa, respectively) was 0.867. Based on Eq. 4 suggesting that the acoustic intensity was proportional to the pressure squared, the intensity transmission coefficient was 0.75. Compared with the theoretical value of 0.82, the acoustic intensity transmission coefficient was 0.07 less than the 3-layer transmission coefficient. The error may be attributed to the difference in acoustic impedance of ultrasonic gel and near-field interference of sound. Accordingly, to stimulate cells at about 300 $mW/{cm}^{2}$, 400 $mW/{cm}^{2}$ of an actual emission of the sound wave was required.

### BrdU incorporation assay

BrdU incorporation assay was utilized to find the most appropriate parameters for LIUS stimulation of hAdMSCs. The procedure of the BrdU incorporation assay is presented in Fig. 5d. Cell proliferation ELISA(Enzyme-linked immunosorbent assay) and BrdU kit (catalog number 11647229001, Roche Applied Science, Germany) showed that the absorbance varied with labeling time. Therefore, it was necessary to establish a linear interval representing the absorbance proportional to the labeling time and the number of cells. After determining the linear interval, the BrdU incorporation assay was performed with a cell density of 1200 cells/well using a labeling time of 16 h. Each assay was performed using a single control. There were five experimental groups (4 wells were for each group) within the area of a LIUS transducer. The average absorbance was calculated from the values of four wells. LIUS stimulation parameters were frequency, intensity, duration, and duty cycle. All experiments were conducted at least thrice or more. Absorbance values were determined 7 times every 10 min starting from time zero.

### Cell culture experiments for 16 days

To validate the effects of LIUS stimulation, two sets of control and stimulation groups were cultured from passages 2 to 6 by recording the number of cells in each passage. Experimental procedures and LIUS stimulation apparatus are shown in Fig. 5e,f. Each group consisted of three 35 mm round Petri-dishes (Catalog number 20035, SPL Life Sciences, South Korea) with 20,000 cells/dish seeded. Cells from three dishes were collected and counted after 4 days of incubation. When subculturing to the following passage, the same number of cells (20,000 cells) were seeded into each dish. Cells were cultured with alpha MEM (Catalog number 32561, Gibco™, USA) supplemented with 8% fetal bovine serum (FBS) (Catalog number 10099, Atlas, USA), antibiotic–antimycotic (Catalog number 15240, Gibco™, USA), hydrocortisone (HC) (Catalog number H0888, Sigma Aldrich, USA), epidermal growth factor (EGF) (Catalog number CHA-EGF, Cha Meditec, South Korea), and fibroblast growth factor (FGF) (Catalog number CHA-BFGF, Cha Meditec, South Korea). The cell culture medium was refreshed every 48 h. After incubation in a humidified cell culture incubator (at 37 °C for 10 min in a 5% CO_2_) cells were washed with normal saline solution (Catalog number 640001081, CJ Healthcare, South Korea), treated with trypLE express solution (Catalog number 12604, Gibco™, USA), and incubated in a cell culture incubator at 37 °C for 10 min with 5% CO_2_. Cells were harvested by centrifugation at 1,500 rotations per minute for 5 min. The number of viable cells was then counted using an automated cell counter (LUNA-II™, Logos Biosystems, South Korea). The temperature of the ultrasound gel was measured after LIUS stimulation to estimate the effect of temperature generation on the surface of the transducer during ultrasonic stimulation.

### Cell isolation

This research has been approved by Jeju National University institutional review board (IRB) (approval number: 2020-061-001) for human cell sourcing. All methods were performed in accordance with the relevant guidelines and regulations. AdMSCs were isolated from 40 ml of adipose tissue donated by a human donor. To separate AdMSCs from adipose tissue, 40 ml of adipose tissue was centrifuged at 1500 rpm for 5 min with normal saline solution diluted 1:1 ratio. After each centrifugation, the upper oil layer was pipetted out and discarded. Then, the remaining adipose tissue was treated with collagenase type I (catalog number 17100017, Gibco™, USA) in a 1:1 ratio and incubated for 30 min in a C shaking incubator at 37 °C. After another centrifugation, tissue was filtered through a 100 μm cell strainer. Purified cells were centrifuged with normal saline solution, and the pellet was then suspended with culture media. Suspended cells were seeded into a T-75 flask with alpha MEM supplemented with 8% FBS, antibiotic–antimycotic, HC, EGF, and FGF, washed with normal saline solution after 2 days of seeding, and the cell culture media was refreshed every day.

### Cell counting

The number of hAdMSCs in the LIUS-stimulated and control group was compared using an automated cell counter (LUNA-II™, Logos Biosystems, South Korea). Cell counter settings were set as dilution factor, 2; size gating, 10–25 mm; noise reduction, 5; live-cell sensitivity, 5; roundness, 30%; declustering level, medium; and focusing method, autofocus. To establish the accuracy of the cell counter, the same number of cells were seeded into seven different wells. The hemocytometer and automated cell counter difference was less than 4%. Each slide of the automatic cell counter was counted four times without overlapping regions. The average values were highly similar to that of the hemocytometer.

### Fluorescence-activated cell sorting (FACS)

The international society for cell & gene therapy mesenchymal stromal cell (ISCT MSC) committee defined multipotent MSCs as plastic adherent, expressing cluster of differentiation (CD) markers of CD 73, 90, and 105 while lacking CD 11b, 14, 19, 34, 45, 79a and HLA-DR, and can differentiate into adipocytes, chondrocytes, and osteoblasts. To roll out that LIUS stimulation does not affect the characteristics of AdMSCs, fluorescence-activated cell sorting (FACS) (BD Biosciences, USA), a derivative of flow cytometry, was adopted. LIUS stimulated and control group AdMSCs from passage 6 were used for FACS analysis, and the data from each test demonstrated 10,000 events. For the purity test, CD markers 34 (Catalog number 555823, Hu CD34 PE-Cy5 581 100Tst, BD Biosciences, USA) and 45 (Catalog number 555482, Hu CD45 FITC HI30 100Tst, BD Biosciences, USA) were used. While PE-Cy^TM^5 and FITC served as the fluorochromes for CD 34 and 45. Moreover, CD markers 73 (Catalog number 561254, Hu CD73 FITC AD2 100Tst, BD Biosciences, USA), 90 (Catalog number 555597, Hu CD90 PE-Cy5 5E10 100ug, BD Biosciences, USA), and 105 (Catalog number 25-1057-42,) CD105 Monoclonal Antibody (SN6) PE-Cyanine7, eBioscience^TM^, USA) were also employed. While FITC, PE-Cy^TM^5, and PE-CY^TM^7 were fluorochromes for CD 73, 90, and 105. Results of both LIUS stimulated, and control groups presented the presence of CD markers of 73, 90, and 105, proving LIUS stimulation does not affect AdMSCs’ characteristics.

### High-throughput cytokine expression assay

A high-throughput human cytokine assay (Human Cytokine Array C2000, Catalogue # AAH-CYT-2000-2, RayBiotech, USA) was performed to screen the cytokines released by LIUS stimulated AdMSCs, and the control group AdMSCs. This assay analyzed 174 cytokines related to the paracrine, autocrine, and endocrine function of the AdMSCs. The cell culture supernatant was utilized to perform a high-throughput human cytokine assay according to the manufacturer’s instructions.

### AdMSCs differentiation and characterization

AdMSCs have a characteristic feature of differentiating into adipogenic, osteogenic, and chondrogenic linage. AdMSCs were cultured for 21 days with DMEM supplemented with 10% FBS, 1 μM dexamethasone (Signa-Aldrich, USA), 500 μM 3-isobutyl-1-methylxanthinacin (Sigma-Aldrich, USA), 10 µg/mL insulin (Signa-Aldrich, USA) and 100 µM indomethacin (TCI, Tokyo, Japan) for adipogenic differentiation. While, for chondrogenic differentation, AdMSCs were cultured for 21 days with StemPro™ chondrogenesis differentiation kit (Gibco). Osteogenic differentiation was carried out by culturing AdMSCs for 21 days with the StemPro chondrogenesis differentiation Kit (Gibco, Waltham, MA, USA). All the supplemented cell culture media were changed after every 2 days. After the AdMSCs differentiation, the resultant cells were examined with Oil Red O stain (Sigma-Aldrich, Burlington, MA, USA)., Alizarin Red S stain (Sigma-Aldrich, Burlington, MA, USA)., and Alcian Blue stain (Sigma-Aldrich, Burlington, MA, USA). for adipogenic, osteogenic, and chondrogenic linage evaluation respectively.

### Statistical analysis

All statistical analysis were performed using the MATLAB software Version 2019a (MathWorks Corp., USA)^47,48^. Statistical values were expressed as mean, SD, and t-test values. The correlation analysis was performed using the ttest2 function in MATLAB software. The ttest2 function tests the null hypothesis that two data samples are derived from populations with the same mean. The significance level was set to be *p* < 0.05 for all cases.

### Ethical approval

Written consent was obtained from all participants. The Institutional Review Board approved the study protocol of Jeju National University (Approval Number: 2020-061-001).

## Supplementary Information

Supplementary Information.

## Data Availability

The datasets used and analyzed during the current study are available from the corresponding author upon reasonable request.
