# Supplementary material for: Production enhancement of human adipose-derived mesenchymal stem cells by low-intensity ultrasound stimulation
Source: Sci Rep. 2022 Dec 21;12:22041. doi: 10.1038/s41598-022-24742-0 (PMC9772213; doi:10.1038/s41598-022-24742-0)
Supplement: Supplementary file 1 — Supplementary Information. [file 41598_2022_24742_MOESM1_ESM.docx]

| **Fixed parameters**  Intensity: 300 mWcm^-2^  Duty cycle: Continuous wave  Stimulation time: 10 min | | **Fixed parameters**  Frequency: 5 MHz,  Duty cycle: Continuous wave  Stimulation time: 10 min | | **Fixed parameters**  Frequency: 5 MHz,  Intensity: 300 mWcm^-2^,  Stimulation time: 10 min | | **Fixed parameters**  Frequency: 5 MHz,  Intensity: 300 mWcm^-2^,  Duty cycle: Continuous wave | |
| --- | --- | --- | --- | --- | --- | --- | --- |
| **Stimulation**  **Conditions** | **Temperature**  **°C** | **Stimulation**  **Conditions** | **Temperature**  **°C** | **Stimulation**  **Conditions** | **Temperature**  **°C** | **Stimulation**  **Conditions** | **Temperature**  **°C** |
| 4 MHz | 28 | 100 mWcm^-2^ | 24.5 | 20% | 25 | 10 min | 25 |
| 5 MHz | 25 | 300 mWcm^-2^ | 26 | 40% | 25 | 20 min | 26 |
| 6 MHz | 38.5 | 500 mWcm^-2^ | 29 | 60% | 25 | 30 min | 28 |
| 9 MHz | 42 | 700 mWcm^-2^ | 42 | 80% | 25.5 | 10 min x2 per day | 24.5 |
| 10 MHz | 28.5 | 900 mWcm^-2^ | 45 | 100% | 26 | 10 min x3 per day | 25 |

**Supplementary Information**

**Production enhancement of human adipose-derived mesenchymal stem cells by low-intensity ultrasound stimulation**

**Table S1.**
